# Adaptive Impulse Reconstruction of Seismic Signals Induced by TBM Drilling Noise via CEEMDAN-Assisted MDD Interferometry

**DOI:** 10.3390/s26041115

**Published:** 2026-02-09

**Authors:** Lei Zhang, Guowei Zhu

**Affiliations:** 1 College of Geoscience and Surveying Engineering, China University of Mining and Technology (Beijing), Beijing 100083, China; bqt2000202025@student.cumtb.edu.cn; 2 State Key Laboratory for Fine Exploration and Intelligent Development of Coal Resources, China University of Mining and Technology (Beijing), Beijing 100083, China

**Keywords:** tunnel ahead prospecting, TBM drilling noise, seismic interferometry, impulse-like reconstruction, CEEMDAN mode screening, multidimensional deconvolution

## Abstract

Tunnel ahead prospecting is important for reducing construction risks associated with faults, fractured zones, and cavities ahead of the tunnel face, but controlled active-source surveys are often impractical during continuous TBM operation. TBM drilling-noise records provide persistent passive excitation; however, strong nonstationarity and narrowband tonal contamination can hinder stable retrieval of interpretable impulse-like responses. We propose an adaptive impulse reconstruction algorithm that couples CEEMDAN-based mode screening with MDD interferometry. CEEMDAN screening suppresses quasi-stationary tonal components while preserving coherent propagation-related wavefields, producing effective signals suitable for interferometric processing. The MDD stage is stabilized using band-limited inversion, phase-only whitening, and a multi-reference strategy. Numerical experiments with a 3D elastic tunnel model indicate that the proposed workflow yields a more compact and laterally coherent virtual-source gather than correlation-based baselines (CC and PHAT-CC) and single-reference deconvolution interferometry, supporting reflection-oriented interpretation beyond simple wavelet compression. Field measurements from an operating TBM tunnel, together with a hammer-impact benchmark, are consistent with the feasibility of the workflow under real tunneling conditions and with physically plausible moveout behavior in the reconstructed gathers.

## 1. Introduction

Unexpected geological anomalies (e.g., faults, fractured zones, karst cavities and water-bearing structures) ahead of the tunnel face may cause severe construction delays and safety risks during mechanized tunneling. To reduce such uncertainties, active-source ahead-prospecting techniques have been widely applied in practice (e.g., Tunnel Seismic Prediction, TSP, using explosive/hammer sources in boreholes), providing reflection-based indications of adverse structures in front of the excavation face [1,2,3,4,5]. However, these methods often require dedicated source operations, interrupting excavation or demanding additional working space and manpower, which may limit their timeliness and deployment efficiency in continuously advancing tunnel boring machine (TBM) tunnels.

In contrast, TBM rock-breaking signals naturally provide persistent vibration energy during excavation and thus offer an attractive opportunity for near-real-time seismic probing without additional active sources. Early studies demonstrated that tunnel seismic data can be acquired while drilling by treating TBM noise as a passive excitation [6], and subsequent developments further improved interface prediction and wavefield reconstruction for practical tunneling scenarios [7,8]. Recent work has continued to explore TBM-noise-based forward prospecting and scatterer localization, highlighting the potential of passive/operational sources for tunnel-scale sensing [9,10,11,12,13]. Nevertheless, compared with controlled hammer or explosive shots, TBM rock-breaking signals usually exhibit strong spectral line components, time-varying nonstationarity, and complex mixtures of direct, scattered and reflected wavefields, making stable retrieval of interpretable impulse-like responses challenging [14].

Seismic interferometry provides a theoretical foundation for reconstructing Green’s functions from uncontrolled or operational sources by exploiting wavefield correlations in space and time [15,16,17]. Correlation-based interferometry has been widely used for ambient-noise applications and engineering monitoring, yet its results may be affected by source directivity, incomplete illumination and non-ideal wavefield assumptions [18,19]. To mitigate these issues, interferometry by deconvolution has been introduced to reduce source-signature imprint and improve temporal focusing [20]. Furthermore, multidimensional deconvolution (MDD) extends the deconvolution concept by incorporating multi-receiver constraints, effectively compensating for the point-spread effect and reducing the dependence on a single reference trace, thereby yielding more robust and better-balanced responses [21,22,23]. These characteristics are particularly attractive for TBM tunnel observations, where reference-channel quality may vary, and the recorded wavefield can be severely contaminated by machine-induced oscillations.

However, for TBM rock-breaking data, directly applying interferometric deconvolution or MDD to raw recordings may still lead to unstable solutions when the input is dominated by persistent oscillatory components and narrow-band interference. Therefore, it is necessary to adaptively suppress TBM-related line spectra and enhance effective wavefield components prior to interferometric solving, so that the subsequent MDD inversion is fed with signals that are more interpretable and physically consistent. Empirical mode decomposition (EMD) and its noise-assisted variants provide a fully data-driven way to handle nonlinear and nonstationary signals [24,25,26]. In particular, ensemble-based schemes such as Ensemble Empirical Mode Decomposition (EEMD)/complete ensemble empirical mode decomposition (CEEMDAN) and their improved versions have demonstrated strong capability in alleviating mode mixing and extracting meaningful intrinsic mode functions (IMFs) under noisy conditions [27]. This motivates a combined strategy: using CEEMDAN-style adaptive decomposition to construct an effective input for MDD-based interferometry, aiming at a more stable impulse-like reconstruction from TBM rock-breaking signals.

In this paper, we propose an adaptive impulse reconstruction algorithm for TBM rock-breaking seismic signals by integrating CEEMDAN-based mode screening with MDD-assisted interferometry. The algorithm first performs noise-assisted EMD decomposition and multi-domain IMF selection to suppress oscillatory interference and preserve coherent wavefield components, and then applies MDD interferometry to obtain an impulse-like virtual shot gather with improved interpretability. The proposed algorithm is validated using both numerical modeling and a field experiment in an operating TBM tunnel, demonstrating its effectiveness and improving the interpretability and practical potential of TBM rock-breaking seismic records for ahead-of-face probing.

## 2. Materials and Methods

### 2.1. TBM Drilling-Noise Seismic Signals and Numerical Validation Setup

This study focuses on seismic signals generated by TBM rock breaking and aims to improve their interpretability for tunnel-ahead prospecting. To provide a controlled benchmark for evaluating the proposed CEEMDAN-assisted MDD interferometric impulse reconstruction, we constructed a 3-D elastic forward-modeling framework and generated synthetic three-component seismic records under two excitation types: a controlled active-source benchmark and a TBM-equivalent drilling-noise excitation. 

#### 2.1.1. Numerical Simulation Scheme and Stability

We performed 3-D elastic forward modeling using an explicit velocity–stress formulation on a staggered grid. Spatial derivatives were evaluated with a second-order finite-difference stencil, and the wavefield was advanced in time with an explicit scheme. Boundary reflections were suppressed by applying a convolutional perfectly matched layer (CPML) on all sides of the model.

The time step was selected according to a conservative Courant–Friedrichs–Lewy (CFL) setting based on the maximum wave speed in the model *v*_max_ and the minimum grid spacing Δ_min_, i.e., $C=v_{\max}\Delta t/\Delta_{\min}$. With Δ*t* = 0.02 ms, Δ_min_ = 1 m, and *v*_max_ = 5200 m/s, the Courant number is *C* = 0.104, providing a conservative stability margin for the adopted simulations. No numerical blow-up was observed in the simulated wavefields. The key numerical settings are listed in Table 1.

#### 2.1.2. Numerical Model and Observation Geometry

**Figure 1:**
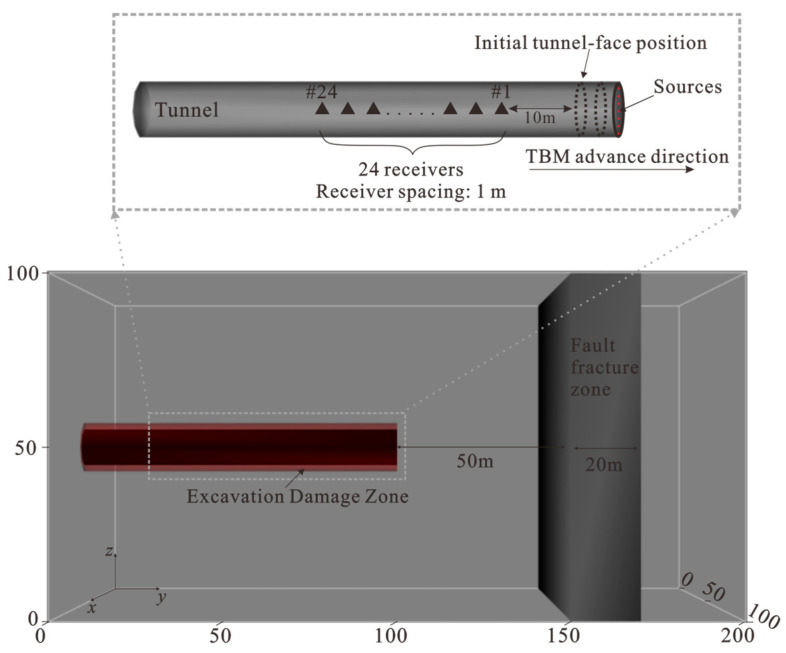
3-D elastic model and acquisition geometry used for both the controlled active-source benchmark and the TBM-equivalent drilling-noise simulations. The model contains a tunnel cavity with an EDZ and a fractured zone ahead of the face (front-boundary offset: 50 m; thickness: 20 m). The receiver array is deployed along the tunnel sidewall. The TBM-equivalent source advances from *y* = 100 m to *y* = 109 m (10 positions).

A 3-D elastic model with dimensions of 100 m × 200 m × 100 m was constructed, in which intact granite was assumed as the background medium. The tunnel axis is aligned with the y-direction, and a cylindrical excavation cavity is embedded at the model center (Figure 1). To account for excavation-induced loosening and enhanced scattering near the boundary, an excavation-damaged zone (EDZ) was included as a 2 m-thick annulus surrounding the tunnel [28]. A fractured zone was further placed ahead of the tunnel face to represent a distributed low-velocity heterogeneity. In the reference configuration (Figure 1), the leading interface of the fractured zone is located 50 m in front of the face, and the zone thickness is 20 m. The corresponding elastic parameters assigned to the host rock, tunnel, EDZ, and fractured zone are listed in Table 2.

A single receiver line composed of 24 three-component receivers was deployed along the tunnel sidewall at *x* = 56 m and *z* = 50 m, with receiver positions spanning *y* = 67–90 m at a spacing of 1 m. For the controlled active-source benchmark, forward modeling was performed at the initial tunnel-face position (#1) under the same acquisition geometry. For the TBM-equivalent drilling-noise excitation, the source position was stepped along the tunnel axis to form ten locations from *y* = 100 m to *y* = 109 m. This compact one-sided geometry is used throughout the numerical validation.

#### 2.1.3. Source Design and Benchmark Dataset

**Figure 2:**
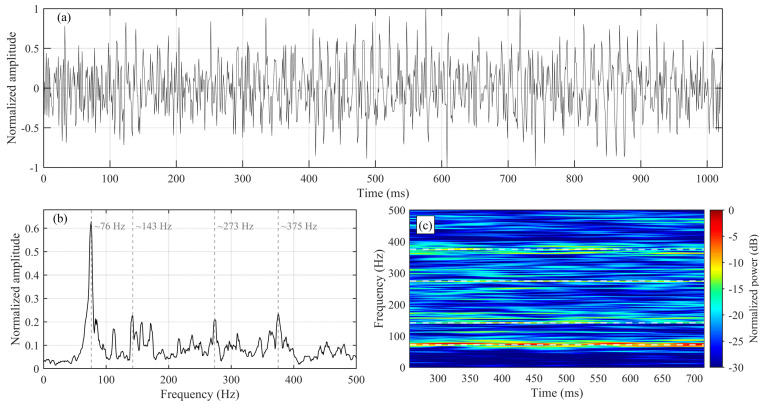
TBM−equivalent drilling-noise excitation prescribed in the forward simulations: (**a**) Normalized time-domain waveform. (**b**) Normalized amplitude spectrum. (**c**) Time–frequency spectrogram.

Two excitation types are simulated for a controlled validation. The target case adopts a TBM-equivalent drilling-noise excitation, which is quasi-continuous and nonstationary in time (Figure 2a). Its spectrum exhibits persistent narrowband peaks with surrounding energy spread (Figure 2b), with dominant components around ~76 Hz, ~143 Hz, ~273 Hz, and ~375 Hz. The corresponding time–frequency map shows long-lasting tonal lineations at these bands (Figure 2c), consistent with the superposition of narrowband components related to TBM rotation or rock-breaking cycles and broadband fluctuations commonly observed in drilling-noise records [9,10].

At each TBM-equivalent source position, a distributed point-source set was used to emulate spatially distributed loading and radiation near the tunnel face. Specifically, 10 grid points were randomly selected in the vicinity of the tunnel-face center, and the same waveform was injected at these points in a Y-dominant manner. The random selection was performed independently for each position to reflect the spatial variability of the TBM-induced loading.

As a controlled reference, an impulsive benchmark dataset was generated under the same model and receiver layout by exciting the model with a band-limited Ricker wavelet (*f*_0_ = 200 Hz) at the initial tunnel-face position (#1). This impulsive benchmark provides clear arrivals and reflection timing and serves as a consistent reference for assessing interpretability improvements brought by interferometric impulse reconstruction.

### 2.2. TBM-Adaptive CEEMDAN Mode Screening and Effective-Signal Construction

TBM drilling-noise records are strongly nonstationary and often contain persistent narrowband tonal interference and pronounced amplitude fluctuations. These features can degrade trace-to-trace consistency and hinder subsequent interferometric processing. We therefore apply CEEMDAN trace-wise to obtain a multiscale representation and then retain physically meaningful modes to construct an effective signal for later interferometric reconstruction.

#### 2.2.1. Energy-Calibrated CEEMDAN Decomposition

Let $S_{i}^{\left(s\right)}(t)$ denote the time-domain signal at receiver *i* for the *s*-th source position (or time segment). CEEMDAN decomposes each trace into a set of IMFs and a residual trend,

(1) $$S_{i}^{\left(s\right)}(t)=\overset{K}{\underset{k=1}{\sum }}c_{k,i}^{\left(s\right)}(t)+r_{i}^{\left(s\right)}(t)$$

where $c_{k,i}^{\left(s\right)}(t)$ denotes the *k*-th IMF and $r_{i}^{\left(s\right)}(t)$ is the final residual.

TBM drilling-noise records commonly exhibit strong energy fluctuations. To make the ensemble construction more consistent under such variability, we use an energy-calibrated noise-injection amplitude. Specifically, the perturbation level is scaled by a local energy measure,

(2) $$\sigma_{n,i}^{\left(s\right)}(t)=\beta \sqrt{E_{i}^{\left(s\right)}(t)}$$

where *β* is a tuning coefficient and $E_{i}^{\left(s\right)}(t)$ is estimated from a sliding-window mean-square energy of $S_{i}^{\left(s\right)}(t)$. With this scaling, perturbations are strengthened in low-energy intermittent intervals to improve statistical robustness, while being reduced during high-energy impact segments to better preserve transient details and stabilize the subsequent mode screening. The key CEEMDAN parameters used in this study are summarized in Table 3.

#### 2.2.2. TBM-Adaptive Mode Screening and Effective-Signal Construction

The CEEMDAN decomposition yields a pool of candidate IMFs, whereas only a subset exhibits propagation-related characteristics associated with TBM rock breaking. Modes dominated by persistent tonal components, slow trends, or receiver-dependent unstable oscillations can reduce cross-trace consistency and introduce artifacts in subsequent interferometric processing. We therefore perform TBM-adaptive mode screening by jointly enforcing complementary criteria in the frequency, time, and multi-trace domains, and then construct an effective signal by summing only the retained IMFs.

(i) Frequency-domain relevance with anti-tonal control.

For each IMF, we compute the in-band energy ratio *r*_band_ within the target band $\left[f_{\min},f_{\max}\right]$ relative to its total spectral energy. IMFs with negligible in-band contribution ($r_{\mathrm{band}}<r_{\mathrm{band},\min}$) are rejected. For IMFs that satisfy the in-band requirement, we further quantify tonal dominance using the peak-to-band energy ratio *R*_peak_ (energy of the strongest spectral peak divided by the total in-band energy). Line-dominated IMFs ($R_{\mathrm{peak}}>R_{\mathrm{peak},\max}$) are excluded to prevent persistent TBM line spectra from dominating the retained set.

(ii) Time-domain transience and energy concentration.

To favor arrival-like components, each IMF is evaluated by its excess kurtosis κ (computed from the time-domain samples) and by a short-time energy concentration ratio *p_E_*, defined as the maximum fraction of IMF energy captured in a sliding window of length *T*_conc_. IMFs with weak impulsiveness and poor concentration ($\kappa <\kappa_{\min}\;\mathrm{or}\;p_{E}<p_{E,\min}$) are rejected because they tend to represent long-lasting oscillations or trends and blur interferometric products.

(iii) Cross-trace consistency within a shot gather.

Physically meaningful components should be coherent across receivers under the compact one-sided array. We compute a multi-trace coherency curve using a sliding window *T*_coh_ and select coherent samples by a quantile threshold *q*_coh_. For each IMF, we then calculate the coherency-energy ratio *C*_coh_, defined as the mean IMF energy within coherent samples divided by that within incoherent samples. IMFs with insufficient cross-trace consistency ($C_{\mathrm{coh}}<C_{\mathrm{coh},\min}$) are rejected.

After screening, the retained IMFs are more concentrated on propagation-related arrivals associated with TBM rock breaking, rather than quasi-stationary tonal interference or slow trends. The effective signal is constructed as follows:

(3) $$S_{\mathrm{eff},i}^{\left(s\right)}(t)=\underset{k\in K_{i}^{\left(s\right)}}{\sum }c_{k,i}^{\left(s\right)}(t)$$

It serves as the default input for the subsequent interferometric processing. The retained index set $K_{i}^{\left(s\right)}$ is stored to ensure full reproducibility of the effective-signal construction. The key parameters for mode screening and effective-signal construction are summarized in Table 4.

### 2.3. Seismic Interferometry for Wavefield Retrieval from TBM Drilling Noise

The energy-calibrated CEEMDAN decomposition and TBM-adaptive mode screening construct effective signals that suppress quasi-stationary tonal components to a certain extent and enhance propagation-related consistency across receivers. Building on these effective signals, we compute virtual-source responses using interferometric operators. In this study, we report three baseline reconstructions, cross-correlation (CC), its phase-weighted variant (PHAT-CC), and deconvolution interferometry (DC), and then focus on multidimensional deconvolution interferometry (MDD) as the primary operator for wavefield retrieval from TBM drilling-noise data.

Let $s_{\mathrm{eff},i}^{\left(s\right)}(t)$ denote the effective signal at receiver *i* for the *s*-th source position (or time segment), and let *r* denote a selected reference receiver.

#### 2.3.1. Cross-Correlation Interferometry

We compute a CC estimate between *i* and *r* from the effective signals $s_{\mathrm{eff},i}^{\left(s\right)}(t)$ and $s_{\mathrm{eff},r}^{\left(s\right)}(t)$ [16]. The CC response is defined as follows:

(4) $${\hat{g}}_{ir}^{\mathrm{CC}}\left(t\right)=\frac{1}{N_{s}}\overset{N_{s}}{\underset{s=1}{\sum }}\int s_{\mathrm{eff},i}^{\left(s\right)}\left(\tau \right)s_{\mathrm{eff},r}^{\left(s\right)}\left(\tau +t\right)d\tau$$

where *N_s_* is the number of source positions (or segments).

Because TBM records can remain influenced by residual narrowband peaks even after effective-signal construction, we additionally consider a phase-weighted correlation baseline (PHAT-CC) [19]. In the frequency domain, let $C_{ir}^{\left(s\right)}(\omega )=S_{\mathrm{eff},i}^{\left(s\right)}(\omega )S_{\mathrm{eff},r}^{\left(s\right)}(\omega {)}^{*}$ denote the cross-spectrum for the *s*-th realization. The PHAT-CC response is computed as

(5) $${\hat{g}}_{ir}^{\mathrm{PHAT}\text{-}\mathrm{CC}}(t)=F^{-1}\left\{\frac{1}{N_{s}}\overset{N_{s}}{\underset{s=1}{\sum }}\frac{C_{ir}^{\left(s\right)}(\omega )}{|C_{ir}^{\left(s\right)}(\omega )|+\epsilon }\right\}$$

where $F^{-1}\{\cdot \}$ denotes the inverse Fourier transform and *ϵ* is a small constant for numerical stability. This phase-weighting down-weights amplitude-dominated tonal peaks and places more emphasis on phase consistency, which is more directly tied to relative timing and moveout patterns.

#### 2.3.2. Deconvolution Interferometry

DC is introduced to obtain a more compact virtual wavelet by normalizing each channel spectrum with that of the reference receiver [20]. Let $s_{\mathrm{eff},i}^{\left(s\right)}(\omega )$ denote the Fourier transform of $s_{\mathrm{eff},i}^{\left(s\right)}(t)$. The stabilized DC estimate in the frequency domain can be written as follows:

(6) $${\hat{G}}_{ir}^{\mathrm{DC}}\left(\omega \right)=\frac{\sum_{s=1}^{N_{s}}s_{\mathrm{eff},i}^{\left(s\right)}(\omega )s_{\mathrm{eff},r}^{\left(s\right)}(\omega {)}^{*}}{\sum_{s=1}^{N_{s}}{\left|s_{\mathrm{eff},r}^{\left(s\right)}\left(\omega \right)\right|}^{2}+\epsilon }$$

where ${\left(\cdot \right)}^{*}$ denotes complex conjugation and *ϵ* is a stabilization term to avoid numerical instability. In our implementation, DC is computed within the working band $\left[f_{\min},f_{\max}\right]$ and shares the same band and taper settings as the other operators for consistent comparison. While DC often yields a sharper wavelet than correlation baselines, it can remain sensitive to the quality and spectral characteristics of the reference channel.

#### 2.3.3. Multidimensional Deconvolution Interferometry

While DC relies on a single reference trace, MDD interferometry formulates wavefield retrieval as a multichannel deconvolution problem so that the constraints can be distributed across multiple reference receivers [21,22,23]. For each angular frequency *ω* within the working band, we arrange the effective signal spectra into a receiver–realization matrix $S(\omega )\in C^{N_{r}\times N_{s}}$, whose (*i*, *s*)-th entry corresponds to $S_{\mathrm{eff},i}^{\left(s\right)}(\omega )$. We select a compact set of reference receivers R with $N_{\mathrm{ref}}=|R|$, and form the corresponding reference submatrix $S_{R}(\omega )\in C^{N_{\mathrm{ref}}\times N_{s}}$ by extracting the rows indexed by R.

Under an interferometric forward model, the observed matrix is approximated by

(7) $$S(\omega )\approx G(\omega )S_{R}(\omega )$$

where $G(\omega )\in C^{N_{r}\times N_{\mathrm{ref}}}$ represents an impulse-like virtual-source response matrix. We estimate G(ω) by solving a Tikhonov-regularized least-squares problem, leading to

(8) $$\hat{G}(\omega )=S(\omega )S_{R}(\omega {)}^{H}{\left(S_{R}(\omega )S_{R}(\omega {)}^{H}+\lambda I\right)}^{-1}$$

where ${\left(\cdot \right)}^{H}$ is the conjugate transpose, *λ* is a stabilization parameter, and I is the identity matrix. Finally, $\hat{G}(\omega )$ is transformed back to the time domain to obtain virtual-source gathers for subsequent interpretation.

#### 2.3.4. TBM-Adaptive Robustification Strategies

TBM drilling-noise records are commonly contaminated by persistent narrowband tonal components and time-varying amplitudes. These features can distort spectral estimates and weaken waveform focusing in correlation- and deconvolution-based interferometry. To keep the comparisons consistent across operators, we apply the same working band $\left[f_{\min},f_{\max}\right]$ and taper settings to all methods and use phase-only whitening where appropriate. In this study, $\left[f_{\min},f_{\max}]=[50,\;500\right]$ Hz is adopted as a common working band (consistent with Section 2.2.2), which we treat as the effective frequency range of TBM rock-breaking excitation for subsequent interferometric processing. In our implementation, the following measures are adopted:

(i) Band-limited inversion with a short taper.

The processing is restricted to the working band $\left[f_{\min},f_{\max}\right]$, and a short symmetric time-domain taper is applied prior to the Fourier transform. This combination reduces spectral leakage and boundary effects and yields more stable spectral estimates within the working band. By limiting the operation to the band where the effective signal is reliable, out-of-band components are prevented from dominating subsequent interferometric products.

We use [50, 500] Hz throughout for cross-operator comparability, and apply a short symmetric taper of 5–15 ms (default 10 ms). If attenuation is strong or the SNR above 400–500 Hz is low, the high-frequency end can be conservatively reduced, but the same band should be kept for all operators within a given comparison.

(ii) Phase-only whitening within the working band.

To limit the influence of persistent tonal peaks, we apply phase-only whitening in $\left[f_{\min},f_{\max}\right]$, which reduces spectral contrast while preserving the phase information that governs relative timing and moveout. For the correlation baseline, this corresponds to PHAT-weighting of the cross-spectrum (PHAT-CC). For the deconvolution-type operators (DC and MDD), the same phase-only whitening can be applied to the effective-signal spectra before forming the normal-matrix terms. This normalization down-weights amplitude-dominated tonal peaks and makes the result depend more on phase consistency across realizations.

Phase-only whitening is applied only within [50,500] Hz. We recommend enabling it for TBM drilling-noise records with prominent tonal peaks; if tonal peaks are weak, it can be disabled for a closer-to-amplitude reconstruction.

(iii) Multi-reference constraints.

A multi-reference set R is used instead of a single reference trace so that the constraints are distributed across multiple receivers. This design reduces the dependence on any single channel and provides more stable solutions when individual traces are affected by local coupling variations or intermittent spectral anomalies. In the least-squares sense, multiple references provide redundant constraints, which reduces variance in $\hat{G}(\omega )$ when individual reference traces are imperfect.

We recommend using 4–8 reference traces (or roughly 20–35% of the receiver channels) that are spatially distributed and avoid visibly unstable or poorly coupled sensors. Using too few references increases sensitivity to individual traces, whereas using many highly similar references often yields diminishing returns.

(iv) Conditioning-aware regularization.

The stabilization parameter *λ* in Equation (8) is selected to balance temporal focusing and numerical stability. During parameter selection, the conditioning of $S_{R}(\omega )S_{R}(\omega {)}^{H}$ is monitored as an auxiliary indicator to mitigate ill-conditioned inversions and spurious artifacts. A larger *λ* effectively damps poorly conditioned spectral components, whereas a smaller *λ* favors temporal focusing when the system is well conditioned.

We select *λ* from a small candidate set on a logarithmic scale and recommend λ∈[0.03,0.2] (default 0.1 for this study). In practice, *λ* can be chosen as the smallest value that avoids obvious ringing or instability in the retrieved gathers while remaining consistent with the conditioning statistics reported in Section 3.4.

### 2.4. Evaluation Metrics

**Figure 3:**
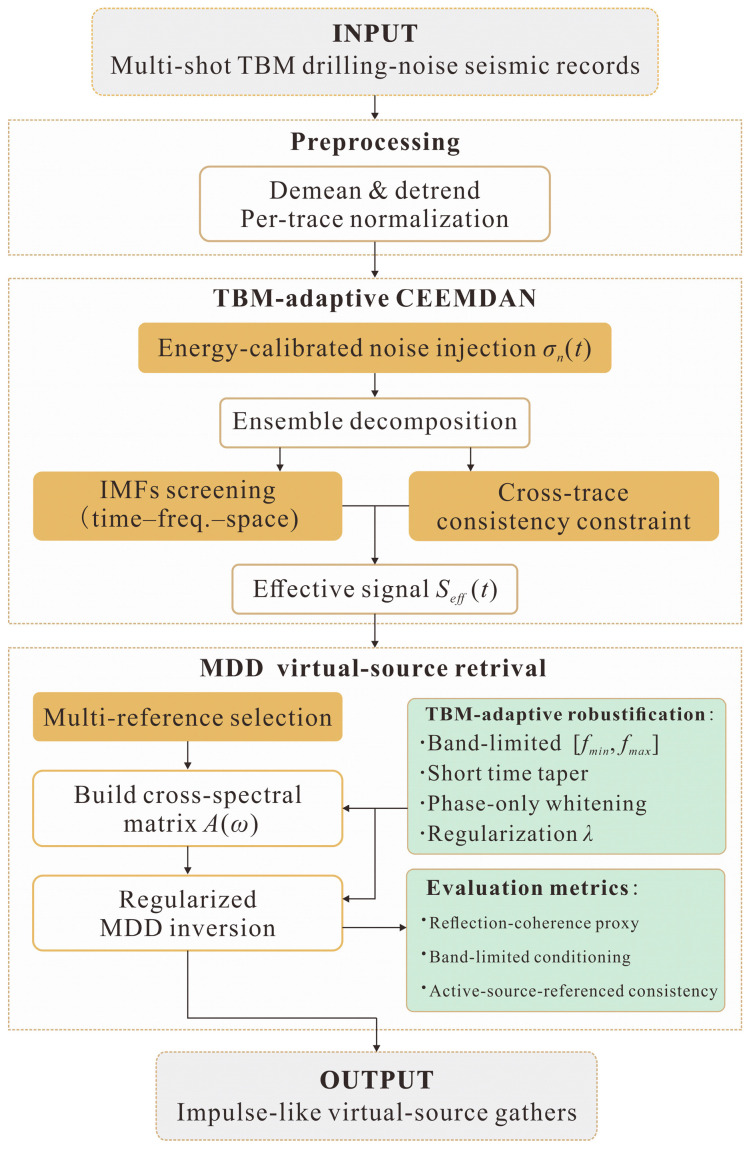
Schematic overview of the proposed CEEMDAN-assisted MDD interferometric algorithm for impulse-like signal reconstruction.

Based on the effective signal construction in Section 2.2 and the interferometric operators in Section 2.3, Figure 3 summarizes the proposed algorithm. To evaluate the retrieved impulse-like gathers from complementary perspectives, we use three metrics throughout this study: (i) a reflection-coherence proxy to quantify the relative prominence of reflection-window energy over the early-time/background level, (ii) an active-source-referenced consistency index to quantify the agreement with controlled active-source gathers and thus assess the interpretability of reflection-related patterns, and (iii) a band-limited conditioning statistic to characterize the numerical stability of the MDD inversion.

(i) Reflection coherence proxy.

To quantify whether the reconstructed gathers exhibit more prominent reflection-window energy relative to the early-time level, we introduce a coherence proxy based on the envelope RMS measured within two time windows. Let ${\tilde{G}}_{i}(t)$ denote the reconstructed trace at receiver *i* (*i* = 1, …, *N_r_*).

We then define

(9) $$R_{\mathrm{coh}}=\frac{\frac{1}{N_{r}}\sum_{i=1}^{N_{r}}{\mathrm{RMS}}_{t\in W_{\mathrm{refl}}}\left(E\{{\tilde{G}}_{i}(t)\}\right)}{\frac{1}{N_{r}}\sum_{i=1}^{N_{r}}{\mathrm{RMS}}_{t\in W_{\mathrm{early}}}\left(E\{{\tilde{G}}_{i}(t)\}\right)}$$

where E{⋅} is the envelope operator (magnitude of the Hilbert transform), and $W_{\mathrm{early}}$ and $W_{\mathrm{refl}}$ represent the early-time and reflection-analysis windows, respectively. Larger *R*_coh_ indicates that energy becomes more concentrated within the reflection window relative to the early-time level.

(ii) Active-source-referenced consistency.

To assess the interpretability of reflection-related patterns, we measure the agreement between the reconstructed gathers and a controlled active-source reference. Let $G_{\mathrm{act}}(i,t)$ denote the gather from controlled active source excitation, and $G_{m}(i,t)$ denote the gather reconstructed by method m∈{CC,PHAT−CC,DC,MDD} for each given TBM source position group. Because the two gathers may not share the same time zero, we first align them by their picked direct-wave arrivals and then compute the similarity within the reflection-analysis window $W_{\mathrm{refl}}$ as

(10) $$C_{\mathrm{act}}^{\left(m\right)}=\frac{{\langle \mathrm{vec}\left(E\{G_{\mathrm{act}}\}\right),\mathrm{vec}\left(E\{G_{m}\}\right)\rangle }_{W_{\mathrm{refl}}}}{{\left\|\mathrm{vec}\left(E\{G_{\mathrm{act}}\}\right)\right\|}_{W_{\mathrm{refl}}}{\left\|\mathrm{vec}\left(E\{G_{m}\}\right)\right\|}_{W_{\mathrm{refl}}}}$$

where E{⋅} is the envelope operator and vec(⋅) stacks all traces within the reflection-analysis window $W_{\mathrm{refl}}$ into a single vector. A larger value of $C_{\mathrm{act}}^{\left(m\right)}$ indicates stronger agreement of reflection-related patterns with the active source reference.

(iii) Band-limited conditioning.

For MDD interferometry, the stability of the frequency-domain inversion is governed by the normal matrix $A(\omega )=S_{R}(\omega )S_{R}(\omega {)}^{H}$. We quantify its conditioning by

(11) $$\kappa \left(\omega \right)=\mathrm{cond}\left(A\left(\omega \right)\right)=\frac{\sigma_{\max}\left(A\left(\omega \right)\right)}{\sigma_{\min}\left(A\left(\omega \right)\right)}$$

where *σ*_max_ and *σ*_min_ are the largest and smallest singular values, respectively. A band-limited summary over the processing band $\left[f_{\min},f_{\max}\right]$ is reported as

(12) $${\bar{\kappa }}_{\mathrm{band}}=\frac{1}{N_{f}}\underset{\omega \in \Omega_{\mathrm{band}}}{\sum }\kappa (\omega )$$

where $\Omega_{\mathrm{band}}$ denotes the discrete frequency samples within $\left[f_{\min},f_{\max}\right]$. In general, a lower ${\bar{\kappa }}_{\mathrm{band}}$ indicates a better-conditioned system and a more stable MDD solution.

## 3. Results

### 3.1. Baseline Raw Records: Active-Source Versus TBM-Equivalent Excitation

**Figure 4:**
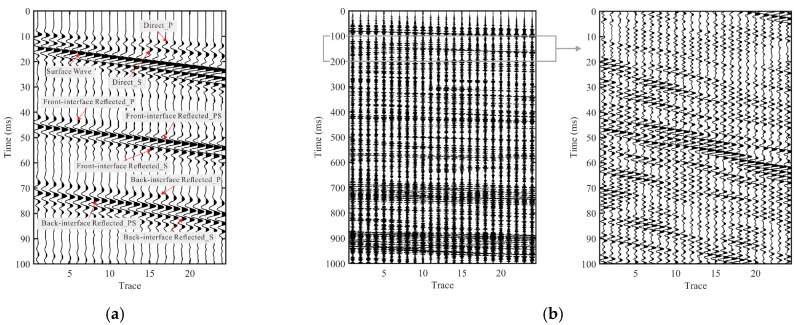
Baseline comparison of raw shot gathers (Y component) under two source conditions for the fracture-zone model. (**a**) Active impulsive source. (**b**) TBM-equivalent drilling-noise excitation shown over 0–1000 ms, with a zoomed view of 100–200 ms indicated by the box.

Figure 4 compares the baseline raw Y-component shot gathers of the fracture-zone model under an active impulsive source (Figure 4a) and a TBM-equivalent drilling-noise excitation (Figure 4b). In the active-source case, the gather exhibits clear and coherent moveout patterns, enabling straightforward recognition of the main wave groups (Direct_P, Direct_S, and the surface wave) as well as reflection-related event bands associated with the front and back interfaces (Front-interface Reflected_P/PS/S and Back-interface Reflected_P/PS/S). These annotated arrivals provide a convenient timing and interpretation reference for subsequent comparisons.

In contrast, the TBM-equivalent gather is characterized by more persistent and superposed energy with reduced lateral coherence, making reflection-related events less distinguishable. To highlight this behavior, Figure 4b presents the gather induced by TBM-equivalent drilling-noise excitation in a longer time window (0–1000 ms) together with a zoomed view of 100–200 ms (marked by the box). Importantly, this 100–200 ms interval is adopted as the fixed analysis window for subsequent TBM-equivalent numerical experiments.

### 3.2. Effective-Signal Construction by TBM-Adaptive CEEMDAN

Following the fixed 100–200 ms analysis window adopted for TBM-equivalent records (Figure 4b), we apply the proposed TBM-adaptive CEEMDAN screening trace by trace and construct an effective gather $S_{\mathrm{eff},i}^{\left(s\right)}(t)$ by summing the retained IMFs.

**Figure 5:**
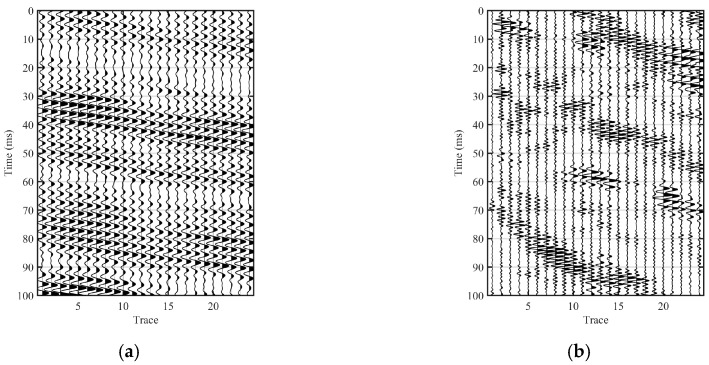
CEEMDAN-based construction of the effective gather and the corresponding residual gather for the TBM-equivalent drilling-noise excitation dataset (Y component), shown within the fixed analysis window. (**a**) Effective gather *S*_eff_ constructed by TBM-adaptive CEEMDAN screening. The retained modes (IMF5–IMF8 in this example) are selected according to the joint multi-domain criteria in Table 4. (**b**) Residual gather *S*_res_ composed of the discarded components (the non-retained IMFs) and the residual term.

Figure 5 presents the CEEMDAN result for the TBM-equivalent drilling-noise excitation record. Figure 5a shows the CEEMDAN-screened effective gather $S_{\mathrm{eff},i}^{\left(s\right)}(t)$ constructed by TBM-adaptive CEEMDAN screening, while Figure 5b shows the corresponding residual gather $S_{\mathrm{res},i}^{\left(s\right)}(t)=S_{\mathrm{raw},i}^{\left(s\right)}(t)-S_{\mathrm{eff},i}^{\left(s\right)}(t)$. Compared with the raw gather in Figure 4b, the effective gather preserves the major propagation-related event bands and makes them more traceable across receivers, thereby improving interpretability. Nevertheless, the wavelet in *S*_eff_ is still strongly controlled by the TBM-equivalent drilling-noise excitation, exhibiting a relatively broad main lobe and pronounced coda/dragging; it is therefore not yet an impulse-narrowed input for reliable event interpretation, motivating the subsequent interferometric reconstruction for further impulse-like sharpening and robustification.

As a complementary view of the screening outcome, the residual gather in Figure 5b is dominated by more dispersed, background-like components and lacks the stable event bands and lateral continuity seen in Figure 5a, indicating that the rejected IMFs mainly capture non-target persistent/unstable oscillations. Based on these observations, *S*_eff_ is used as the default input for the interferometric reconstructions in the following sections.

### 3.3. Interferometric Reconstruction Results: CC, PHAT-CC, DC, and MDD Interferometry

**Figure 6:**
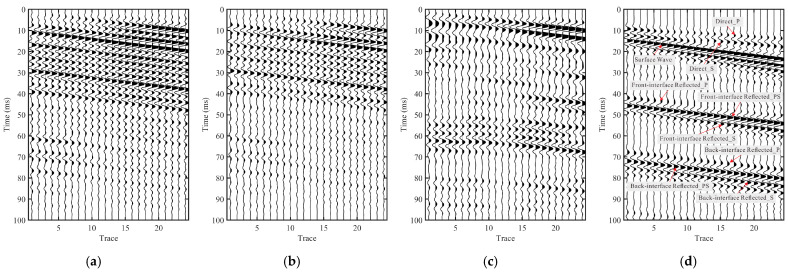
Comparison of interferometric reconstructions from the effective signals *S*_eff_ (Y component). (**a**) Conventional cross-correlation baseline (CC). (**b**) Phase-weighted cross-correlation baseline (PHAT-CC). (**c**) Stabilized deconvolution interferometry (DC) under a good-reference setting. (**d**) Multidimensional deconvolution interferometry (MDD). In (**d**), key wave groups and reflection-related event bands are annotated following the controlled active-source reference in Figure 4a to facilitate interpretive comparison.

Figure 6 compares four interferometric reconstructions obtained from the effective signals *S*_eff_ (Y component), including the conventional cross-correlation baseline (CC, Figure 6a), its phase-weighted variant (PHAT-CC, Figure 6b), stabilized deconvolution interferometry (DC, Figure 6c), and multidimensional deconvolution interferometry (MDD, Figure 6d). All panels are displayed with consistent plotting settings to enable direct visual comparison.

In the CC result (Figure 6a), the response is dominated by relatively broad wavelets and noticeable trace-to-trace variability. Coherent energy can be identified around the early-time window (approximately 10–20 ms), whereas later arrivals appear more diffuse and show limited lateral continuity, which makes reflection-related bands difficult to track reliably.

Compared with CC, PHAT-CC (Figure 6b) suppresses amplitude-dominated narrowband influence and yields a more phase-consistent gather, most clearly in the early-time window. The early arrivals become cleaner and more stable across traces, but reflection-related bands remain only partially continuous and can still be affected by residual coda-like oscillations.

The DC reconstruction (Figure 6c) further compresses the wavelet relative to the correlation baselines, with stronger energy concentration. However, localized dominance and residual oscillatory artifacts may still appear on several traces, indicating that the deconvolution outcome remains sensitive to the spectral characteristics of the reference channel even under a good-reference setting, where the reference trace is selected as the channel exhibiting stable coupling and the highest early-time signal-to-noise ratio within the analysis window.

In contrast, the MDD-based reconstruction (Figure 6d) provides the most interpretable virtual gather among the four methods. Multiple reflection-related event bands are distinctly traceable across the array, and the associated wavelets appear more compact with reduced trailing oscillations. When compared with the controlled active-source benchmark (Figure 4a), the prominent reflection bands in the MDD gather exhibit comparable relative arrival times with respect to the direct arrivals and show clearer moveout trends across traces. By comparison, the correlation baselines and DC retain more dispersed or locally distorted reflection-related bands.

### 3.4. Quantitative Assessment of Interpretability and Retrieval Stability

**Figure 7:**
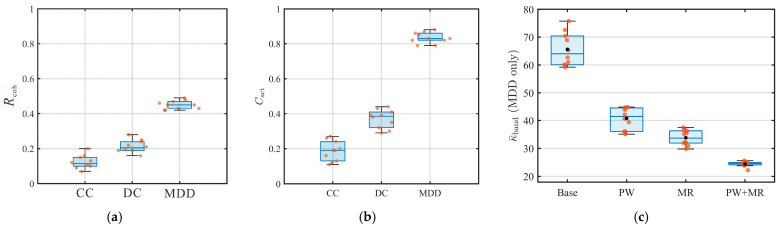
Position-wise statistics of reflection prominence, active-source agreement, and MDD conditioning. (**a**) Reflection coherence proxy *R*_coh_ for correlation-based operators (CC and PHAT-CC), DC, and MDD. (**b**) Active-source-referenced consistency *C*_act_ between the controlled active-source gather and the reconstructed gathers (CC, PHAT-CC, DC, and MDD). (**c**) Band-limited conditioning statistic ${\bar{\kappa }}_{\mathrm{band}}$ of the MDD normal system under different robustification settings: Base (no whitening, *N*_ref_ = 1), PW (phase-only whitening, *N*_ref_ = 1), MR (no whitening, multi-reference), and PW + MR (phase-only whitening with multi-reference). Dots represent individual TBM source-position groups (*n* = 10); boxplots summarize the corresponding distributions.

To quantitatively evaluate the retrieval quality of the interferometric reconstructions, we use the three metrics defined in Section 2.4 and summarize the results in Figure 7. For *R*_coh_ and *C*_act_, statistics are computed position-wise across the available TBM source position groups (*n* = 10). For ${\bar{\kappa }}_{\mathrm{band}}$, conditioning is evaluated for the MDD normal system under different robustification settings, with position groups providing the sample distribution. All measurements use the same fixed time windows $W_{\mathrm{early}}=[0,\;20]$ ms and $W_{\mathrm{refl}}=[30,\;100]$ ms.

As summarized in Figure 7a, the reflection-coherence proxy *R*_coh_ increases from CC to PHAT-CC and then to DC, and reaches the highest level for MDD. This indicates that the envelope energy becomes progressively more concentrated within the reflection analysis window relative to the early-time level. The boxplots and position-wise scatter plots further suggest a tighter distribution for MDD, consistent with a more stable enhancement of reflection-window energy across TBM source-position groups.

The interpretability of the reconstructed gathers is further assessed using the active-source-referenced consistency *C*_act_ (Figure 7b). A consistent ranking is observed across the correlation-based operators and the deconvolution-based operators: PHAT-CC improves over CC, DC yields a higher median *C*_act_, and MDD achieves the highest median with a relatively compact interquartile range. Notably, DC shows a wider spread than PHAT-CC, which is consistent with the reference sensitivity of deconvolution-type estimates—spectral division may amplify intermittent spectral anomalies on the reference trace—whereas PHAT-CC emphasizes phase consistency. This trend is consistent with the waveform comparison: the MDD reconstructed impulse-like gather (Figure 6d) more closely reproduces the event-band continuity and moveout patterns of the controlled active-source benchmark (Figure 4a) than the baseline operators (Figure 6a–c).

In addition, the numerical stability of the MDD inversion is quantified by ${\bar{\kappa }}_{\mathrm{band}}$. As shown in Figure 7c, both phase-only whitening (PW) and the multi-reference strategy (MR) reduce ${\bar{\kappa }}_{\mathrm{band}}$ relative to the baseline configuration, and their combination (PW + MR) yields the lowest values, suggesting a better-conditioned normal system and a more stable inversion across position groups.

### 3.5. Algorithm Validation in an Operating TBM Tunnel

To further verify the practical applicability of the proposed algorithm under real tunneling conditions, we conducted a field experiment in an operating TBM tunnel, where seismic data were acquired using the same receiver array under two excitation conditions: (i) TBM rock-breaking excitation and (ii) an active hammer-impact benchmark. The hammer-impact record serves as an impulsive reference, enabling a direct and intuitive comparison in terms of waveform compactness, lateral coherence, and overall interpretability of the retrieved virtual-source gathers.

**Figure 8:**
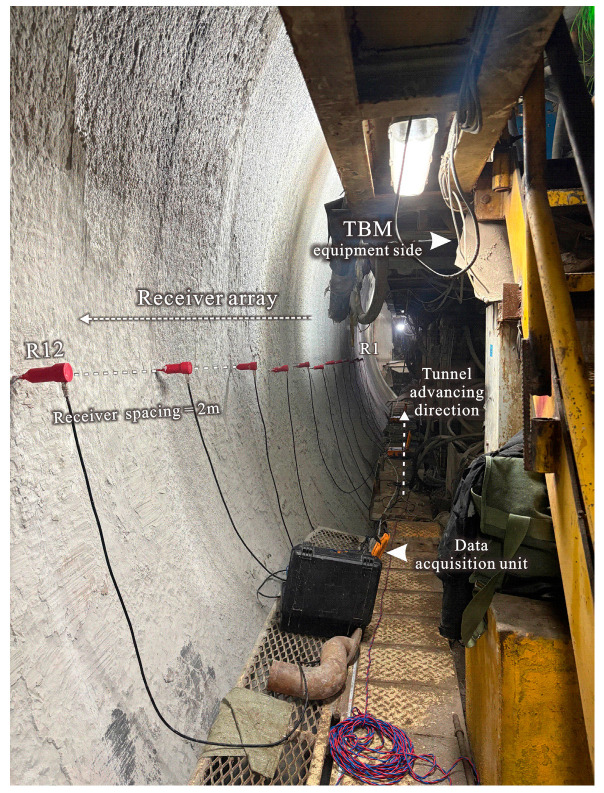
Field acquisition geometry in the operating TBM tunnel.

As illustrated in Figure 8, a single-sided linear array consisting of 12 receivers with a spacing of 2 m was deployed along the tunnel sidewall. To ensure stable coupling and reduce sensor-to-sensor variability, the receivers were installed in pre-drilled boreholes and fixed using gypsum filling, providing robust contact with the surrounding rock mass. The advancing direction of the tunnel and the TBM equipment side are also marked to document the acquisition geometry.

**Figure 9:**
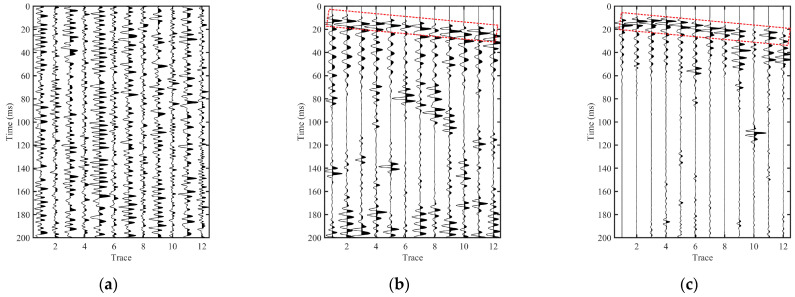
Field-data comparison under TBM rock-breaking excitation and hammer-impact benchmark (Y component). (**a**) Raw passive record induced by TBM rock breaking. (**b**) Impulse-reconstructed virtual-source gather obtained from the raw TBM rock-breaking record. (**c**) Active hammer-impact shot gather was acquired with the same receiver array. (The red dashed box highlights the main direct-wave arrival band).

Figure 9 compares the field records before and after impulse reconstruction. The raw TBM rock-breaking record in Figure 9a is dominated by persistent background oscillations and pronounced ringing, which obscures coherent moveout trends and complicates phase tracking. After applying the proposed impulse reconstruction, the retrieved virtual-source gather in Figure 9b becomes noticeably more compact, with energy concentrated into a clearer main-arrival band; the direct-wave moveout highlighted by the red dashed box also exhibits improved continuity and is easier to follow across the array. In comparison, the hammer-impact record in Figure 9c naturally shows impulsive and compact arrivals, providing a meaningful benchmark for field validation. Overall, the impulse-reconstructed gather exhibits waveform features more consistent with the hammer-impact benchmark, suggesting improved interpretability of field TBM drilling-noise records in practical environments.

It is also observed that, compared with the hammer-impact data, the reconstructed gather still retains noticeable energy over a longer traveltime range. This is consistent with the stronger and more continuous nature of TBM rock-breaking excitation, which is typically low-frequency dominant; such low-frequency components attenuate more slowly in tunnel-surrounding rock, potentially benefiting longer-range propagation.

To further assess the physical plausibility of the reconstructed moveout structure, we estimate the apparent direct-wave velocity from the main arrival band in Figure 9b and compare it with that derived from the hammer-impact record in Figure 9c. The apparent velocity is computed by multi-trace averaging as follows:

(13) $$v_{\mathrm{app}}=\frac{1}{N-1}\overset{N}{\underset{i=2}{\sum }}\frac{\left(i-1\right)\Delta r}{t_{i}}$$

The estimated apparent velocity from the impulse-reconstructed gather is $v_{\mathrm{app}}^{\mathrm{IR}}$ = 3355 m/s, which is close to that obtained from the hammer-impact record $v_{\mathrm{app}}^{\mathrm{HAM}}$ = 3056 m/s. This value falls within the expected range for body-wave propagation in tunnel surrounding rock, indicating that the reconstructed virtual-source gather not only exhibits improved waveform compactness but also preserves physically reasonable moveout behavior.

## 4. Discussion

### 4.1. CEEMDAN Screening for Interferometry-Ready Effective Signals

Deconvolution-type interferometry can be sensitive to inputs that contain persistent tonal interference and strong time variability. In such cases, mode mixing becomes more likely, and the retrieved gathers may be biased toward the reference trace. Variational mode decomposition (VMD) has been used to isolate narrowband components from nonstationary vibration signals, and benchmark tests suggest that it can offer robust mode identification at a lower computational cost than CEEMDAN [29].

In TBM tunneling, however, the wavefield is not purely tonal. Propagation-related transients are intermittent. They coexist with direct, scattered, and reflected contributions. For this reason, our objective is not only to isolate narrowband tones. We also need to preserve propagation-consistent transients that are suitable for interferometric retrieval.

We therefore adopt CEEMDAN as a front-end step to construct an effective signal, rather than to perform generic denoising. Its data-adaptive multiscale IMFs enable targeted screening against components that are inconsistent with coherent, propagation-related wavefields. The retained IMFs are selected to support cross-trace coherence and physically plausible transients, while suppressing quasi-stationary tones and incoherent fluctuations. We also avoid strong band-pass filtering before CEEMDAN, because aggressive pre-filtering can reshape intrinsic scale separation and shift IMF allocation, weakening physical interpretability. Instead, frequency-domain relevance and anti-tonal control are enforced during screening within the working band $\left[f_{\min},f_{\max}\right]$. The interferometric stage then operates within the same working band and applies the stabilization measures described in Section 2.3.4, so that CEEMDAN focuses on adaptive mode separation while interferometry focuses on stable wavefield retrieval.

### 4.2. Reflection Interpretability Beyond Wavelet Compression

To evaluate whether the proposed algorithm improves reflection interpretability, rather than merely narrowing a virtual wavelet, we combine visual inspection with complementary quantitative indicators to summarize gather-level event-band behavior (continuity, stability, and traceability). The reflection-coherence proxy *R*_coh_ describes the relative prominence of energy in the reflection-analysis window compared with an early-time/background baseline, and it characterizes whether reflection-relevant energy becomes more concentrated and band-like. After alignment by direct arrivals, the active-source-referenced index *C*_act_ provides a physics-based check against the controlled active-source benchmark by assessing whether event-band continuity and moveout trends are consistent with that reference.

Under this evaluation perspective, the differences among the four operators mainly reflect different sensitivities to reference dependence and narrowband tonal disturbance. CC is inherently reference-based and can be vulnerable to illumination imbalance and tone-dominated spectra [15,16,17,18]. PHAT-CC down-weights amplitude-dominated narrowband peaks by emphasizing phase consistency and often stabilizes timing and moveout, but residual correlation-type fluctuations may remain [19]. DC can further compress the wavelet, yet it is more sensitive to the spectral characteristics and stability of the chosen reference trace, so unfavorable references may introduce ringing or localized dominance [20]. In contrast, MDD distributes constraints across receivers through a multichannel inverse formulation, reducing single-reference dominance and supporting more interpretable late-time event bands under complex TBM-equivalent drilling-noise excitation [21,22,23].

### 4.3. TBM-Adaptive Robustification for MDD: Mitigating Reference Sensitivity and Improving Stability

**Figure 10:**
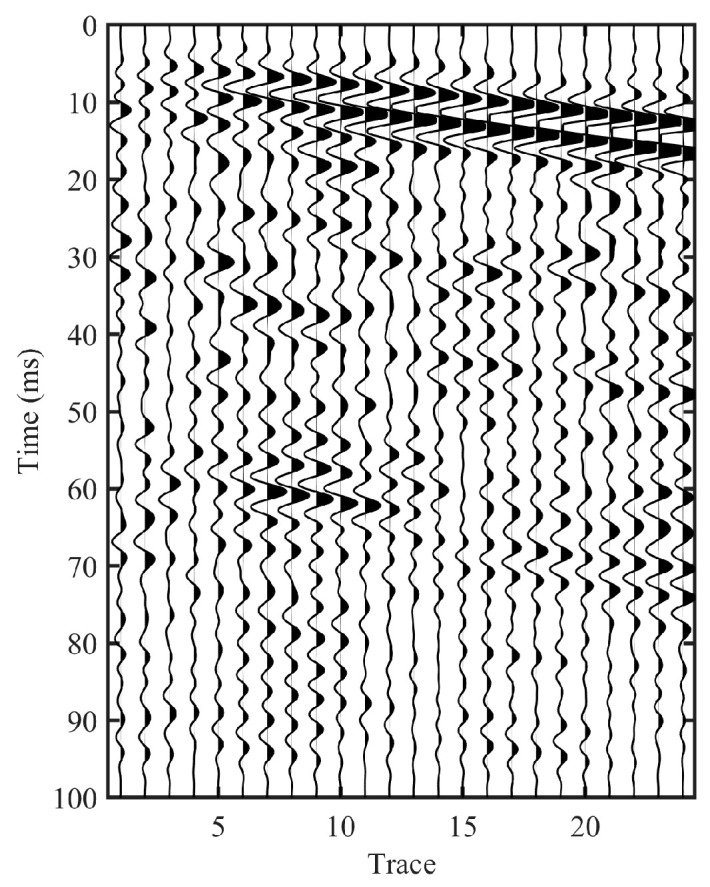
Reference sensitivity of DC under TBM-equivalent drilling-noise excitation with prominent tonal components. The DC-reconstructed gather obtained with a degraded reference illustrates how reference-related spectral/phase instability can imprint localized dominance and ringing-like artifacts, obscuring late-time event-band continuity and weakening interpretability.

In deconvolution-type interferometry, reference selection remains a practical bottleneck. Even after CEEMDAN suppresses quasi-stationary tonal components, a single reference trace may still carry residual line peaks or exhibit spectral/phase instability. Under single-reference normalization, these traits can be amplified and imprinted onto the retrieved gather, which helps explain the pronounced degradation of DC in the illustrative case shown in Figure 10 as reference quality deteriorates. This observation motivates the use of MDD, which distributes constraints across channels and reduces the chance that any single trace dominates the solution.

For MDD, robustness is closely tied to the conditioning of the band-limited normal system. Two complementary strategies are used to address common failure modes in interferometry based on TBM drilling-noise records. Phase-only whitening (PW) reduces excessive spectral contrast associated with narrowband line components, making the inversion less prone to being driven by a few dominant peaks. The multi-reference strategy (MR) reduces reference dependence by distributing constraints across multiple reference traces. Used together, PW primarily targets spectral dominance, while MR targets reference sensitivity. This combination provides a practical route to more stable late-time retrieval under tonal and nonstationary excitation. The associated improvements are consistent with the quantitative conditioning and interpretability measures summarized in Section 3.4.

### 4.4. Practical Implications for Risk Mitigation and Applicability Limits

During excavation, TBM drilling-noise records provide persistent passive observations with much denser temporal sampling than controlled active-source surveys. Yet the raw data are often dominated by tonal components and nonstationary background vibrations, which undermines interpretability and repeatable reflection recognition. By producing virtual-source gathers that are more compatible with reflection-oriented analysis, the proposed method enables coherent late-time events to be evaluated using consistent windows and objective measures. This improved interpretability supports earlier recognition of potentially adverse features ahead of the face and informs risk-aware construction decisions.

CEEMDAN-assisted MDD is computationally heavier than CC/PHAT-CC and DC because it combines ensemble-based decomposition with a band-limited frequency-domain inversion. The workflow is, however, well-suited to parallel computing and windowed processing. For long continuous TBM records, low-cost screening can be used to locate candidate intervals, after which CEEMDAN+MDD is applied only to the selected segments. With short windows and parallel resources, this design supports near-real-time or quasi-online operation. In practice, the additional overhead is most meaningful in tonal, nonstationary noise conditions where correlation- and DC-based gathers remain difficult to interpret, and stable reflection-oriented patterns are needed.

Reconstruction quality can be site-dependent and is influenced by attenuation, scattering strength, and velocity heterogeneity. In strongly attenuative or highly fractured conditions, more conservative retention criteria and stronger stabilization are often beneficial, whereas in clearer propagation environments, a broader working band and weaker constraints may be sufficient. The recommended ranges reported in this study are guided by empirical observations and stability diagnostics on the available dataset. Broader evaluation under different operating conditions and geological regimes would therefore be beneficial, together with case-specific adjustment of key settings, including effective-band selection, screening thresholds, reference strategy, and regularization strength. In addition, the present study validates the method primarily at the gather level; coupling the reconstructed gathers with imaging and interpretation analyses in future studies would help quantify practical resolution and decision-support benefit.

## 5. Conclusions

This study proposes a TBM-adaptive impulse reconstruction algorithm for TBM drilling-noise seismic records by combining CEEMDAN-based mode screening with MDD interferometric retrieval. Based on the comparative results with CC, PHAT-CC, and DC, as well as field validation in an operating TBM tunnel, we draw the following conclusions:

1. CEEMDAN-based screening suppresses quasi-stationary tonal components and constructs an effective signal with improved cross-trace consistency for subsequent interferometric processing;
2. Compared with CC, PHAT-CC, and DC, MDD reduces dependence on a single reference trace and yields more interpretable propagation-related event patterns in the retrieved gathers;
3. The TBM-adaptive robustification, implemented via phase-only whitening and a multi-reference strategy, improves inversion stability and mitigates reference-dominated artifacts;
4. Field measurements in an operating TBM tunnel provide evidence that the proposed algorithm is feasible under real tunneling conditions and can produce a virtual-source gather with a physically plausible travel-time structure.

These results improve the interpretability and stability of TBM drilling-noise seismic records, thereby enhancing the efficiency and reliability of tunnel seismic ahead-of-face prospecting.

## Figures and Tables

**Table 1:** Key numerical modeling settings.

Item	Value
CPML thickness	*n*_pml_ = 20 grid points
Grid spacing	Δ*x* = Δ*y* = Δ*z* = 1 m
Time sampling	Δ*t* = 0.02 ms
Record length (active)	*T_act_* = 100 ms
Record length (TBM)	*T_t_*_bm_ = 1000 ms

**Table 2:** Elastic parameters assigned to each unit in the 3-D simulations.

Unit/Region	*V_p_* (m/s)	*V_s_* (m/s)	*ρ* (kg/m^3^)	Description
Host rock	5500	3200	2700	Intact surrounding rock mass
Tunnel	340	0	1.3	Cylindrical tunnel cavity
EDZ	4000	2300	2500	2 m-thick annular zone surrounding the tunnel
Fractured zone	3200	1800	2400	Fractured-zone anomaly ahead of the face

Note: The tunnel cavity was represented using air-like properties for numerical purposes. The cavity boundary was treated as traction-free in the elastic solver.

**Table 3:** Key parameter settings for the energy-calibrated CEEMDAN decomposition.

Item	Symbol	Value	Note
Energy calibration coefficient	*β*	0.2	Energy-scaled perturbation in Equation (2).
Local-energy window length	*T_E_*	40 ms	Window length for $E_{i}^{\left(s\right)}(t)$.
Local-energy update step	Δ*T_E_*	2 ms	Update step for the sliding-window energy estimate.
Ensemble size	*N*_ens_	200	Number of ensemble realizations.
Max sifting iterations	*I*_max_	2000	Upper bound on CEEMDAN sifting iterations
Maximum number of IMFs	*K*_max_	10	Upper bound on extracted IMFs per trace.

**Table 4:** Key screening parameters for TBM-adaptive mode screening and effective-signal construction.

Item	Symbol	Value	Note
Target frequency band	$\left[f_{\min},f_{\max}\right]$	[50, 500] Hz	Working band for screening.
Minimum in-band energy ratio	*r*_band,min_	0.01	Drop negligible in-band IMFs
Tonal-dominance threshold (peak-to-band)	*R*_peak,max_	1.60	Reject line-dominated IMFs.
Kurtosis threshold (excess)	*κ*_min_	0	Keep non-Gaussian (impulsive) IMFs.
Energy-concentration window length	*T*_conc_	10 ms	Window for *p_E_* evaluation.
Minimum energy-concentration ratio	*p_E,_*_min_	0.20	Require sufficient concentration.
Cross-trace coherency window length	*T*_coh_	5 ms	Window for coherency curve.
Coherency threshold quantile	*q*_coh_	0.60	Coherent samples by quantile.
Minimum coherency-energy ratio	*C*_coh,min_	1.20	Coherent energy must dominate.
Top-*K* safeguard	*K*_top_	3	Prevent empty selection.

## Data Availability

The data presented in this study are available on request from the corresponding author.

## Abbreviations

The following abbreviations are used in this manuscript:

TBM	Tunnel boring machine
EDZ	Excavation damaged zone
CC	Cross-correlation interferometry
DC	Deconvolution interferometry
MDD	Multidimensional deconvolution
PW	Phase whitening
MR	Multi-reference strategy
IMF	Intrinsic mode function
CEEMDAN	Complete ensemble empirical mode decomposition with adaptive noise
